# Nurse-led telehealth intervention effectiveness on reducing hypertension: a systematic review

**DOI:** 10.1186/s12912-022-01170-z

**Published:** 2023-01-17

**Authors:** Maria Kappes, Pilar Espinoza, Vanessa Jara, Amanda Hall

**Affiliations:** 1 Faculty of Health Care Sciences, Nursing School, Universidad San Sebastián, Puerto Montt, Chile; 2 Faculty of Medicine and Science, Universidad San Sebastián, Santiago, Chile; 3 Faculty of Health Care Sciences, Nursing School, Universidad San Sebastián, Santiago, Chile; 4 Heath, Medicine, and Society, Minor Population Health, Biology, Lehigh University, Bethlehem, USA

**Keywords:** Nursing interventions, Telehealth, mHealth, Hypertension, Systematic review

## Abstract

**Background:**

Hypertension is a public health concern for many countries. The World Health Organization has established a global objective to reduce the prevalence of non-communicable diseases, including hypertension, which is associated with cardiovascular disease. Remote nursing interventions can potentially lessen the burden on the healthcare system and promote a healthier population. This systematic review aims to synthesize available evidence on the effectiveness of nursing-led telehealth interventions in reducing blood pressure in hypertensive patients.

**Methods:**

A systematic review was conducted. The search was performed from May to June 2021, in the databases: PubMed, Scopus, Cochrane Library, Web of Science, CINAHL, and ProQuest within 2010–2021 in English, Spanish and Portuguese. Randomized controlled trials and Quasi-experimental studies were considered. This systematic review followed the criteria of the Cochrane Handbook for Systematic Reviews of Interventions, with the support of the PRISMA guidelines and registered in PROSPERO. For critical analysis, the tools of the Joanna Briggs Institute were used.

**Results:**

Of the 942 articles found, six controlled clinical trials and one quasi-experimental study were selected. Different nurse-led interventions (telehealth devices, remote video consultation, calls and email alerts) have demonstrated a significant decrease in blood pressure (especially systolic blood pressure) in the intervention groups. Nurse-led interventions also effect hypertension awareness, self-efficacy, and self-control. Positive effects on lowering cholesterol, consumption of fruits and vegetables, physical activity and adherence to medication were also described.

**Conclusion:**

Nurse-led interventions delivered remotely have a positive effect in lowering the blood pressure of patients with hypertension. Further research is required to support strategies that will deliver the best continuous, quality, and cost-effective nursing care.

**Supplementary Information:**

The online version contains supplementary material available at 10.1186/s12912-022-01170-z.

## Introduction

In 2019, 17.9 million people died from cardiovascular diseases (CVDs) which represents 32% of all global deaths [1]. Hypertension is one of the most important risk factors in the development of CVD [2] and currently, 30% of the world’s population, or approximately one billion people are affected by hypertension [3]. Given hypertension’s relationship to CVD, the World Health Organization (WHO) (2021) established a goal to reduce hypertension by 25% by 2025 [1].

Worldwide, the prevalence of hypertension is higher in low- and middle-income countries, due to the presence of more risk factors associated with diet (consumption of saturated fat and salt) and lifestyle (smoking, sedentary behavior) [2]. The prevalence of complications from hypertension is also greater in low- and middle-income countries, where 50% of mortality from cardiovascular causes occurs between 30 and 69 years, 10 years earlier than in higher-income countries [4]. This problem is especially important in countries that have a rural or dispersed population or those with small numbers of health care providers [5].

## Background

Hypertension, defined as systolic blood pressure (BP) of 130 mmHg or above or diastolic BP of 80 mmHg or above is the main risk factor for CVDs [6], including coronary heart disease, stroke, chronic kidney disease [7], heart failure, arrhythmia, and dementia. Healthy diet (normal salt consumption, low saturated and trans-fat consumption, a high intake of fruits and vegetables), physical activity, and normal weight decrease the risk of developing hypertension. Lifestyle change is a main component in decreasing blood pressure and the related cardiovascular risk [8].

The COVID-19 pandemic has increased barriers of face-to-face interactions between patients and healthcare providers, particularly in primary health settings. To continue to provide care for patients, nurses have begun incorporating different strategies to care for their patients, such as telehealth interventions to monitor and support patients with chronic conditions like hypertension [9, 10].

The concept of telehealth has been in use for more than 30 years, but it was not given a definition by WHO until 2007. The WHO defines telehealth as the deliberate use of communication technologies by healthcare professionals for the diagnosis, treatment and prevention of diseases, as well as the research and continuing education of patients, families and communities where distance between the user(s) and health professionals is a critical factor. According to Mann et al. [11] and Omboni et al. [12], telehealth (also called telemedicine) can be defined as the use of electronic resources to provide efficient and high-quality healthcare. It includes the diagnosis and treatment of patients, as well as the enhancement of patient monitoring techniques such as checking vital signs or reporting symptoms, all with telecommunications technology like smartphones and computers [1, 13]. With the telehealth model, patients do not have to leave their homes to receive a diagnosis or testing results. Telehealth delivery is practiced in all settings and requires the support of different healthcare professionals. The nurse has a vital role in much of this delivery.

The COVID-19 pandemic, declared as such by WHO in March 2020, increased the use of telemedicine to treat patients in many countries [14–16]. Telehealth made it possible to provide diverse care services for patients with chronic diseases. These remote services were fundamental during the pandemic, alleviating the shortage of medical resources and reducing the risk of infection in hospitals and medical centers [17]. In regard to hypertensive patients, telehealth is presented as an opportunity for routine care and continuation of treatment at home even beyond the pandemic [13]. The increased demands for telehealth during the COVID-19 pandemic show the need to prepare nurses to support telehealth and to take the lead in its integration within the healthcare system.

Nursing-led interventions are based on a care delivery model that incorporates assessment, evaluation, education, counseling, treatment and other procedures using a comprehensive nurse-patient (family) approach, with the nursing professional working independently or in interdisciplinary teams [18].

Multiple systematic reviews completed in the last decade have investigated the impact of nursing-led interventions in patients with hypertension, with the incorporation of new roles such as advanced nursing practice [6, 19–21]. Jointly with advanced nursing practices, nursing-led clinical practices emerge, with interventions that, in addition to counseling and education for patient self-management, incorporate the diagnosis and prescription of medications [19]. Patients express a greater satisfaction and adherence to treatment when guided by nursing-led interventions [21], especially compared to medical management alone [22]. A decrease in cardiovascular adverse events and mortality have also been reported with the use of nursing-led interventions [20], where continuity of care has shown a reduction of hospitalizations and readmissions [21]. Since the onset of the COVID-19 pandemic, improvements in remote monitoring of BP coordinated by nurses compared to usual care have been reported [6]. Additionally, the global costs associated with hypertension mortality have increased exponentially in recent years [23]; self-monitoring of BP has been reported as a cost-effective measure to reduce arterial hypertension morbidity and mortality indicators [24].

This systematic review will synthesize recent evidence of the effectiveness of nursing-led telehealth interventions on hypertensive patients to reduce their high blood pressure, actively exploring the potential of telemedicine to solve the current and future health problems.

## Review

### Aims

This study will explore the effectiveness of nurse-led telehealth interventions in adult patients with hypertension. Randomized controlled trials (RCTs) and quasi-experimental studies that explored the effectiveness or impact of telehealth nursing-led interventions on high blood pressure were included in this review. Secondary outcomes of the review included adherence to the antihypertensive therapy and healthy lifestyle behaviors such as smoking, drinking, exercise, and sleep hours.

### Design

A systematic review was performed according to the protocol and extraction form in the Cochrane Handbook for Systematic Reviews of Interventions, version 6.0. [25] The review protocol was registered in PROSPERO (ID: CRD42021262081) and the PRISMA statement was followed to guide this review [26]. Our initial research question was, are nursing-led telehealth interventions effective in lowering blood pressure in hypertensive patients?

### Search methods

The literature search was conducted on PubMed, Scopus, Cochrane Library, Web of Science, CINAHL, and ProQuest from May to July 2021, using the following keywords: Search (Nursing Interventions) AND (Telehealth)) AND (high blood pressure) Filters: Clinical Trial, Randomized Controlled Trial, from 2010 to 2021 Languages English, Spanish and Portuguese.

Reference lists of publications were searched for potentially relevant articles. Grey literature and thesis were also included. The authors consulted a medical librarian in order to help expand the search as noted in Additional file 1: Appendix 1.

#### Inclusion criteria and study selection

To identify all eligible studies, this review considered the following study designs, populations, interventions, and outcome(s):

- Study design: Randomized Controlled trials and quasi-experimental studies.
- Population: Hypertensive adult patients, with or without other comorbidities like diabetes, obesity, and dyslipidemia.
- Interventions: All types of telehealth or phone technologies conducted by nurses, including M-health, telehealth, telemonitoring, virtual interventions, e-coaching, panel monitoring.
- Outcomes: The primary outcome was change in blood pressure, systolic (mmHg) and diastolic pressure (mmHg) or mean arterial pressure (mmHg).

#### Exclusion criteria

Review articles, letters to the editor, book chapters, protocols, and prospective observational designs were excluded. Articles on telehealth interventions done by professionals other than nursing were not considered.

### Search outcomes

According to the inclusion and exclusion criteria, three authors (MK, PE, and VJ) each independently extracted data to check and compare against each other’s work. Microsoft excel (version 16.0) was used to collate the extracted data.

A standardized data extraction form was used to extract data from each study, which included the following criteria: author’s names, country, participants, sample size, intervention/control groups, follow-up period, measurement tools, and results. Rayyan software was used to manage and organize the articles used in this review.

**Fig. 1:**
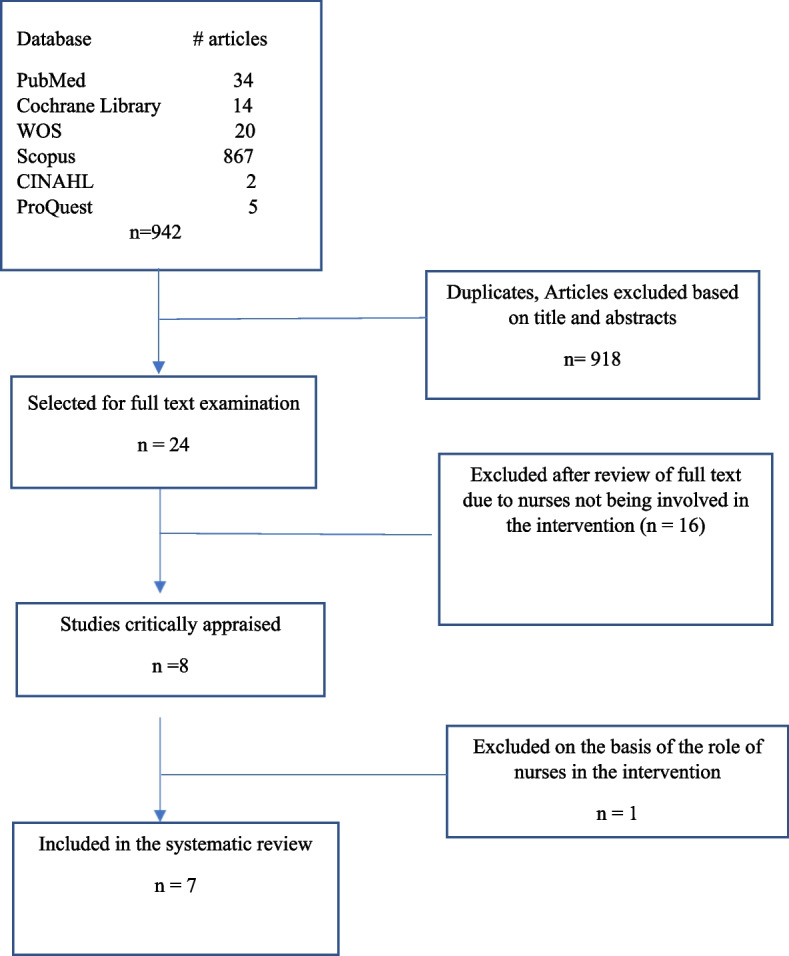
PRISMA article selection flowchart

Nine hundred and forty-two articles were identified with the search strategy. (Fig. 1) In the first stage, duplicate articles and those that did not agree with the objective of the study were excluded based on reading the abstract and title. Of the 24 articles that remained, 16 were excluded because nurses were not involved in the intervention. One article was excluded because it did not describe a nursing-led intervention. Therefore, 7 articles were included in this review.

### Quality appraisal and data extraction

Quality assessment was based on the use of the critical analysis tools of the Joanna Briggs Institute [27]. These tools were selected because each checklist contained an explanation of how to respond appropriately to each item. This tool has also been well evaluated compared with others and is appropriate to use in nursing research [28].

In this review, we used different tools to evaluate controlled clinical trials and quasi-experimental studies (Additional file 2: Appendix 2). These tools consisted of 13 and 9 points, respectively. Each question on the list was answered with “yes,” “no” or “unclear.“ When using the critical analysis tools, we used 2 criteria to decide to include the studies thereby ensuring quality. First, the studies needed to meet at least 60% of the criteria outlined by Chan et al [29] for controlled clinical trials. Second, they also had to meet the following criteria from the tool:

- “Was true randomization used for assignment of participants to treatment groups?”
- “Were treatment groups similar at the baseline?”
- “Were outcome assessors blind to treatment assignment?”
- “Were outcomes measured in the same way for treatment groups?”

The authors required the use these criteria as mandatory elements in order to accept the study for inclusion because they are critical for identifying the risk of bias [25].

Two independent reviewers performed the critical evaluations. In the event of a disagreement between evaluators, a third evaluator was asked to review the study. Following this critical analysis, the studies were included in this review (Fig. 1).

### Synthesis

The data from the included studies information was transferred into an excel table with the items: authors, the aim of the study, population, design, duration, intervention, outcomes, and findings. Although the population and outcomes of the studies were comparable, there was great heterogeneity within the interventions performed, which is why it was impossible to conduct a meta-analysis. Therefore, results are presented in narrative form.

## Results

Through the search strategy, 942 articles were identified. After applying the inclusion and exclusion criteria, 7 articles were included in the final analysis. Figure 1 summarizes the search, identification and selection process of the articles included in this review. The results of the critical evaluation of these 7 studies are summarized in Additional file 3: Appendix 3. Of the 7 studies included in the systematic review, 6 were RCTs and 1 was a quasi-experimental study. A total of 2102 participants completed the studies, and their data were included in the analysis. Age of patients ranged from 54 to 77 years old. The number of female participants was slightly larger than male, and the races included were primarily White, Hispanic and African American. Educational level, familiar arrangements and Body Mass Index (BMI) were reported in all of the studies.

Sample sizes of some of the included studies were calculated using power analysis [6, 30], while the others didn’t mention how they were calculated [31–35]. Wakefield et al. [31] and Bosworth et al. [30] used a random number generator to select participants, in the other 5 studies sampling was done with purpose [6, 32–35]. Four studies used a randomization process to allocate participants into different groups [6, 31–33]. Brennan [32] used a blind recruitment process, as a blind allocating process was used in 3 studies [33–35].

All 7 studies (Table 1) included 1 control group and a different number of intervention groups. Some of them had 1 group [6, 32, 33], some 2 [31, 34], and others 3 groups of participants [30, 35]. While the majority of the studies included single set type of intervention, 2 studies involved mixing intervention content [30, 35]. One study used different levels of monitoring and educational content with all the participants [31], and every study included one control group that received normal care. In terms of follow-up, two studies had a follow-up time of 2 months [6, 35], three studies a 6-month follow-up period [32, 33], one a 12 month follow up [31] and two 18-months follow up [30, 34].

**Table 1:** Articles included in this review

Authors (Year)/Country	Purpose	Participants	Design	Duration	Control Intervention	Intervention	Outcomes (variables)	Findings
Kim, M. (2019)/Korea [35]	To develop long-message services (LMS) and phone-based health-coaching for community-dwelling seniors diagnosed with hypertension and assess the effects of the programs implemented both separately and together.	124 participants aged 65 years or older with hypertension at two senior welfare centers in Seoul, South Korea,	RCT	8 weeks	Usual care	Long-message service and phone-based health coaching	Hypertension self-efficacy, Hypertension self-management, Medication adherence, Hypertension-related knowledge, blood pressure .	Phone-based health-coaching with LMS was effective in improving medication adherence, hypertension self-efficacy, and self-management behaviour and decreasing systolic BP as compared to LMS only. There were also improvements in medication adherence, hypertension related knowledge, hypertension self-efficacy, self-management behaviour, and systolic BP in the LMS group as compared to the control group.
Choi and Kim (2014)/Korea [6]	Develop educational materials and a classification system for remote consultations and home-based healthcare through videoconferencing, manage the blood pressure of patients through a ubiquitous-health (u-health) service, and identify its effects on the blood pressure and level of depression of the service recipients (i.e.,low-income elderly patients with hypertension).	49 male and female hypertension patients older than 65 years of age who were taking an antihypertensive drug, who currently resided in the Permanent Rental Apartments for the low-income in Seoul.	Quasi-Experimental	8 weeks	No further mediation after the installation of the equipment and the initial training	Received blood pressure monitoring as well as inbound–outbound remote video consultation	BP measurement, depression, and healthy lifestyle	The U-health nursing service via videoconferencing made a measurable contribution to a healthier lifestyle by reducing systolic blood pressure levels compared with those who were only monitored for high blood pressure. Therefore, this service is recommended as part of a hypertension management regimen for low-income elderly people as an effective means of nursing intervention.
Cicolini et al. (2014)/Italy [33]	To test the efficacy of a nurse-led reminder program through email (NRP-e) to improve CVD risk factors among hypertensive adults.	198 adult individuals with hypertension	RCT	6 months	Usual care	Received email alerts and phone calls from the nurse care manager regarding healthy lifestyle habits and routine health follow-ups	Number of cigarettes smoked per day, units of alcohol consumed per day, minutes of physical activity per day, number of servings of fruit and vegetables per day, drug use, capillary blood glucose, systolic and diastolic blood pressure, waist circumference, LDL cholesterol, and triglycerides	After 6 months, the following CVD risk factors significantly improved in both groups: body mass index, alcohol and fruit consumption, cigarette smoking, adherence to therapy hours, systolic and diastolic blood pressure, fasting blood glucose, low- density lipoproteins (LDL) and total cholesterol, triglycerides, and physical activity. In the NRP-e group, however, the prevalence of several behaviours or conditions at risk decreased(16%), low fruit consumption (24%), uncontrolled hypertension (61%), LDL (56%), and total cholesterol (40%) increased significantly more than in the control group.
Wakefield et al. (2011)/ Iowa, US [31]	To evaluate the efficacy of a nurse managed home telehealth intervention to improve outcomes in veterans with comorbid diabetes and HTN	257 veterans residing in eastern Iowa and western Illinois, participants had type 2 diabetes and hypertension	RCT	6 months	Usual care	Intervention patients entered BP and BG measurements and responded to standardized questions based on their group assignment. Patients then received appropriate automated responses depending on how they answered the device prompt, that is, correct responses were reinforced, and incorrect responses were reviewed and explained	Depression, Patient adherence, A1C and SBP	Home telehealth provides an innovative and pragmatic approach to enhance earlier detection of key clinical symptoms requiring intervention. Transmission of education and advice to the patient on an ongoing basis with close surveillance by nurses can improve clinical outcomes in patients with comorbid chronic illness.
Hebert, et al. (2012) New York, USA [34]	To test the effectiveness on blood pressure of home blood pressure monitors alone or in combination with follow-up by a nurse manager.	416 Participants were randomized into 3 groups: usual care (176) Blood pressure monitoring group (120) and nurse management + Blood pressure monitoring (120)	RCT	9 and 18 months	Usual care	A one-time educational program about using the home BP monitor, strategies to improve medication adherence and healthy lifestyle done by a nurse manager, plus phone calls, and email alerts to reinforce the information and do a follow-up of participants.	Blood Pressure. Medication adherence, difficulty controlling weight; reducing stress, smoking, alcohol, dietary salt or fat were measured.	The statistically significant changes on BP from intervention groups to usual care were only at 9 months. On the first intervention was − 7.0 mm Hg (Confidence Interval [CI], -13.4 to − 0.6) and in the second was + 1.1 mm Hg (95% CI, -5.5 to 7.8). Changes since baseline in self-reported medication Adherence and smoking did not differ statistically significantly across treatment groups.
Bosworth et al. (2011) North Carolina, USA [30]	To evaluates 3 novel hypertension treatment delivery methods based on home telemonitoring of BP. To determine which of the interventions delivered via telephone was most effective in improving BP control	591 Participants were randomized into 4 groups: 147 In usual care Group, 148 In behavioural management intervention group, 149 In medication management intervention group, 147 In combined intervention group	RCT	6, 12 and 18 months	Usual care	Telemedicine and Home BP Monitoring, behavioural management intervention consisted of 11 tailored health behaviour modules focused on improving hypertension self-management, medication management intervention a nurse provided the physician with a medication change recommendation based on the decision support software, combined intervention: patients received the full dose of each intervention.	The primary outcome of the study was BP control measured at baseline and at 6, 12, and 18 months. They also measured the costs of interventions.	Behavioural management and medication management alone showed significant improvements at 12 months—12.8% (95% confidence interval [CI], 1.6-24.1%) and 12.5% (95% CI, 1.3-23.6%), respectively—but not at 18 months
Brennan et al. (2010)/United States [32]	To determine whether a telephonic nurse disease management (DM) program designed for African Americans is more effective than a home monitoring program alone to increase blood pressure (BP) control among African Americans enrolled in a national health plan.	Self-identified African Americans, age 23 and older, in health maintenance organization plans, with hypertension 954 members recruited, 638 (66.9%) completed the initial assessment.	RCT	6 months	BP monitors and written and nurse-directed phone call instructions to measure their BP at home at regular intervals.	Nurses initiated monthly calls to participants with the goals of improving their hypertension knowledge and supporting lifestyle changes such as smoking cessation, regular exercise, and adherence to the DASH (Dietary Approaches to Stop Hypertension) diet.	Blood pressure, frequency of blood pressure monitoring, number of antihypertension medication classes, healthcare utilization	A nurse DM program tailored for African Americans was effective at decreasing systolic BP and increasing the frequency of self-monitoring of BP to a greater extent than home monitoring alone. Recruitment and program completion rates could be improved for maximal impact.

In terms of intervention, one offered home telehealth devices to improve blood pressure, while a nurse manager reviewed daily data from a blood pressure monitoring device and responses of participants to decide if a closer follow-up, information, reinforcement or referral to health care provider was needed using inbound–outbound remote video consultation [31]. Cicolini el al. [33] and Hebert et al. [34] offered a one-time educational program about using the home BP monitor, strategies to improve medication adherence and healthy lifestyle done by a nurse care manager, plus phone calls, and email alerts to reinforce the information and do a follow-up of participants. Brennan et al. [32] did monthly telephone follow ups of about 15 to 20 min to reinforce hypertension knowledge, medication adherence and support lifestyle changes such as smoking cessation, regular exercise, and healthy diet. Kim [35] and Bosworth et al. [30] used long message service and phone-based health coaching to provide evidence-based recommendations regarding hypertension related behaviors. Kim [35] used an 8-week coaching program and monthly long-distance message, while Bosworth et al. [30] used a behavioral management program of 11 weeks with reinforcing messages, e-mails and calls. Choi’s et al. [6] intervention consisted of a remote consultation twice a week for 8 weeks done by licensed and trained nurses.

Some of the studies’ interventions used algorithms based on guidelines about disease management, lifestyle modification, and treatment, to provide educational content [30–34]. The only one reporting a theoretical framework for the intervention was Kim [35] using Cox’s Interaction Model of Client Health Behavior to develop phone-based health coaching. Only one study did not provide a clear description to support their intervention [6].

Concerning the professional nurses participating in the 7 studies, some were nurses [30], registered nurses [34] with some with previous experience in delivering home telehealth and nursing care management [31, 33], while others were trained licensed nurses [6]. The disease management nurses received training in cardiac care and cultural care competency before the study [32], and registered nurses were required to complete over 40 h of training in phone-based health coaching [35]. Herbert et al. [34] used a trained nurse for the intervention arm without providing details in terms of the type of training or education that was delivered. None of the studies described if the intervention nurses had any postgraduate education such as a BSN, MSN or doctoral degree.

Nurse-led interventions included educational interventions [6, 30, 32–34], such as training the patient to measure blood pressure [6, 30, 32–34], performing patients remote consultation, undertaking video conferences with the participants [30], taking responsibility for phone calls and email [31–35] doing long message service and delivering phone health coaching to participants [35] or using a software application to deliver educational scripts and algorithms [30]. In Herbert et al. [34] study, nurses contacted patient’s clinicians to address medications problems and arrange any prescription changes.

### Effects of interventions on patients’ blood pressure

The Wakefield et al. [31] study compared two remote monitoring intensity levels and usual care in hypertensive patients with type 2 diabetes being treated in primary care. The high-intensity group received a set of messages based on a disease management algorithm programmed into an electronic device focusing on diet, exercise, smoking cessation, foot care, advice for sick days, medications, weight management, preventive care, and behavior modification and lifestyle showed significant improvement (*p* = 0.001) on the blood pressure compared with the low intensity group (not set of messages or algorithm) and the usual care group. The Choi et al. [6] study compared remote video consultation and blood pressure monitoring vs. only blood pressure monitoring. The difference in resulting systolic BP between the control and experimental groups was statistically significant, although the difference in diastolic BP was not statistically significant. The strategy used in the study by Cicolini et al. [33] consisted of an educational program for both groups and was added to the experimental group weekly email alerts and phone calls from a nurse care manager. Systolic and diastolic blood pressure significantly decreased in both groups (all *p* < 0.01) but in the intervened group, obesity, low fruit consumption, total cholesterol and uncontrolled arterial pressure decreased more significantly.

A study conducted by Kim [35] reviewed the efficacy of telephone messages on patients’ blood pressure. Phone-based health-coaching with long message service was effective in decreasing systolic BP as compared to long message service only (*p* < 0.05). In another study, the systolic BP adjusted mean of the intervention group was significantly lower than of the control group (123.6 vs. 126.7, P.0.03) post-intervention, nevertheless, there was no statistically significant difference in diastolic BP between the groups at the end of the intervention [32].

All patients of the intervention arm (3 groups) of Bosworth et al. [30] study used telemedicine and home BP monitoring compared to the usual care group. The improvement in BP control to usual care at 12 months was statically significant in the first group that received behavioral management by nurses with 12.8% (95% CI, 1.6–24.1%; *P* = 0.03) and also on the medication management group with the physician and the nurse working together with 12.5% (95% CI, 1.3–23.6%; *P* = 0.03). Differences on BP with the third intervention (combination of intervention 1 and 2) and usual care were not statistically significant.

Herbert et al. [34] divided participants into three groups, the first involved BP monitoring and registered nurse counseling and telephone follow up for 9 months, the second only received the BP monitor, and the third group received usual care. The statistically significant changes on BP from intervention groups to usual care were only at 9 months. On the first intervention was − 7.0 mm Hg (Confidence Interval [CI], -13.4 to − 0.6) and in the second was + 1.1 mm Hg (95% CI, -5.5 to 7.8).

### Effects of intervention on patient adherence to medication and healthy lifestyle

Adherence to medication was assessed using one question of the Morisky Medication Adherence Scale [36], and improved over time for both groups, although there was no significant difference among them [33]. In the Wakefield et al. [31] study, adherence to the antihypertensive therapy was measured using two scales and reported no significant differences between the 3 groups. Medication adherence in Kim study [35] was measured using a scale developed by Morisky et al. [36] and the results showed significant differences between the 4 groups. An adjusted version of Healthy Lifestyles and Lifestyle Behavior tool were used in the Choi et al. [6] study, and the results indicated that the only significant difference found was in sleep patterns and hobbies. Physical activity was evaluated through a questionnaire previously validated by the authors and was compared between the intervention and control group showing a statistically significantly greater improvement in BMI, alcohol consumption, cigarette smoking, fruit consumption, and physical activity on the intervention arm of the study [33].

## Discussion

The purpose of this systematic review was to explore the effectiveness of nursing-led telehealth interventions on adult patients with high blood pressure. This systematic review indicates that different nurse-led interventions (telehealth devices, remote video consultation, calls and email alerts) demonstrated a significant decrease in blood pressure (especially systolic blood pressure) in intervention groups. Nurse-led interventions also effect hypertension awareness, self-efficacy, and self-control. Positive outcomes related to lowering cholesterol, improving consumption of fruits and vegetables, increased physical activity and adherence to medication were also described.

The findings of the systematic review included different populations, all of them reported interventions guided, managed, and performed by nurses. These results are consistent with a systematic review completed in 2021 which reported that nurse-led interventions provided coordinated interventions that support continuity of care for people with chronic disease [18].

The interventions included in this systematic review were effective primarily in achieving changes in blood pressure and as well as improving patients’ knowledge acquisition related to a healthy lifestyle [30, 32, 33, 35]. When reviewing the content of these interventions, common elements were noted such as the quest for medication adherence and the focus on education in healthy lifestyles habits like food consumption, frequent exercise, smoking cessation, etc. The sources of information delivered to patients through the interventions in this systematic review have also been reported in other studies [32, 37, 38] and were based on nationwide guidelines and evidence regarding a healthy lifestyle and blood pressure management.

The technologies used in the present study included electronic devices to measure blood pressure and telehealth devices to enable data transmission. Some of the studies used personalized messages, videoconferencing and videos with information for patient education, however, the majority used emails and telephone calls to provide efficient and high-quality healthcare. Findings from two systematics reviews that studied the use of mobile applications and multiple media (SMS, email and telephone call) to take care of chronic conditions reported different level of outcomes effectiveness [39, 40] and disparities in the quality of the evidence provided [39].

The results of this systematic review have shown that intensity, defined as the frequency of interactions with the participants or in the amount of material delivered to some groups, resulted in differences in blood pressure results. Interactions with participants during the study showed significant variability among the 7 studies and within the studies. For example, one study sent text messages more than once a day depending of the of the BP results [31], others offered remote videoconferencing consultation twice a week [6], one receive email alerts with a reminder of the compliance with heathy lifestyle and occasional phone calls [33] one had monthly telephone interactions with a specific attention to the cultural aspects of care while improving their hypertension knowledge [32] or nursing health coaching for 30 min once a week [35]. Bosworth’s study [30] used 3 types of alert messages triggered by readings of high BP and those messages included elements of a behavioral management intervention. Herbert et al. [34] used regular telephone follow-up (frequency not reported) to reinforce messages on adherence to medication and healthy lifestyles. Findings from a recent systematic review support the findings of the present study reporting improvements in hypertension self-management behavior and medication adherence using interventions that combined tailored messages, interactive communication, and multifaceted functions [41].

Follow-up time in this systematic review ranged from 6 months to one year and nurses were responsible of sending emails or completing phone calls to patients requesting information regarding BP and/or answering questionnaires. All studies reported a loss of participants during the follow-up which may have influenced the results. Perhaps therapeutic relationship building and communication between nurses and their patients on the telehealth context needs different patterns of interaction to build empathy and rapport to facilitate the continuity of care.

Findings of this systematic review specified the nurse’s background and preparation for each study. Four of the studies reported including experienced nurses in the field of chronic disease management [6, 30, 31, 33], one included a nurse manager [34], two studies highlighted the need to have the nurses go through a special course on cultural competency [32] and formal training on telephone health coaching [35] prior to participating in the study. Other studies have reported that Registered Nurses (RN) have the undergraduate preparation to promote healthy lifestyles, prevent the development of disease and deliver care and follow-up to different health conditions on community heath setting [42, 43], never the less, telehealth content and experiences among different levels of nursing education appeared to remain low [44].

### Implications for nursing

Health services recognize that interventions developed and performed by nurses support hypertensive patients in controlling their disease and preventing complications, especially in primary care and developing countries. Telehealth has been gaining ground recently as an effective and efficient strategy to deliver health care, especially in remote geographic areas, with an insufficient number of health professionals and a lack of specialized care. The COVID-19 pandemic limited nurse-patient interactions in the health center context and forced the exploration of new strategies that would allow continuity of care, especially in patients with chronic diseases such as hypertension. It is in this context that telehealth is emerging with great force. The results of this systematic review will provide a foundation from which to build standardizing successful nurse-led interventions for patients with hypertension, using the technological resources already available in their healthcare centers including computers, telephones, and smart phones to send e-mails, messages and/or telephone calls. It is hoped that some lessons learned from the COVID-19 pandemic will be transformed into a quality and safe care for all, reaching all who need it, regardless of where they are.

### Limitations

There were some limitations to this review. Given the small number of articles found searching the 6 databases, it is possible that the search terms may not have covered all possible terms, though we searched for studies in English, Spanish and Portuguese. As the nurse-led interventions were heterogeneous, especially regarding the type of online resources used, intensity of intervention and follow-up, it was difficult to quantify their benefits and compare one intervention to another. Therefore, most of the results were presented in a narrative summary. Additionally, the effects of the nursing-led interventions delivered remotely might depend upon the nature of the content delivered and the online resources used. The patient’s cultural and educational backgrounds, state of the disease and its complications, and baseline blood pressure level measurements may have influenced the assessment accuracy of nurse -led interventions.

This analysis was limited to a population of adult hypertensive outpatients, and nurse-led interventions may not be able to be generalized to other patients with chronic conditions. More research may be needed to establish a consistent online nurse-led intervention program for patients with hypertension and to test the effectiveness of using different telehealth resources.

## Conclusion

Although nurse -led interventions for the management and control of high blood pressure in primary care are effective, it is important to resolve whether they continue to be effective when using technological resources to deliver this care remotely. With the tools used, the selected studies were of high quality, however, the small number of subjects who were evaluated does not allow for generalization of the results.

After searching the scientific literature from the last 10 years, in multiple databases and different languages, and after critically reviewing all relevant studies, this systematic review analyzed only 7 studies to evaluate the effectiveness of nurse -led interventions in controlling high blood pressure in patients diagnosed with hypertension. This study describes the best evidence for making informed decisions regarding the strategies that will deliver the best continuous, quality, and cost-effective nursing care.

## Supplementary Information

**Additional file 1: Appendix 1.** Search strategy.

**Additional file 2: Appendix 2.** Critical appraisal tools.

**Additional file 3: Appendix 3:** Critical appraisal results for included studies.

## Data Availability

All data generated or analyzed during this study are included in this published article [and its supplementary information files].
