# Supplementary material for: Nurse-led telehealth intervention effectiveness on reducing hypertension: a systematic review
Source: BMC Nurs. 2023 Jan 17;22:19. doi: 10.1186/s12912-022-01170-z (PMC9843665; doi:10.1186/s12912-022-01170-z)
Supplement: Supplementary file 1 — Additional file 1: Appendix 1. Search strategy. [file 12912_2022_1170_MOESM1_ESM.docx]

**Pubmed**

Search

Results: 34

Search: ((Nursing Interventions) AND (Telehealth)) AND (high blood pressure) Filters: Clinical Trial, Randomized Controlled Trial, from 2010 - 2021

(("nursing"[MeSH Terms] OR "nursing"[All Fields] OR "nursings"[All Fields] OR "nursing"[MeSH Subheading] OR "nursing s"[All Fields]) AND ("intervention s"[All Fields] OR "interventions"[All Fields] OR "interventive"[All Fields] OR "methods"[MeSH Terms] OR "methods"[All Fields] OR "intervention"[All Fields] OR "interventional"[All Fields]) AND ("telehealth s"[All Fields] OR "telemedicine"[MeSH Terms] OR "telemedicine"[All Fields] OR "telehealth"[All Fields]) AND ("hypertension"[MeSH Terms] OR "hypertension"[All Fields] OR ("high"[All Fields] AND "blood"[All Fields] AND "pressure"[All Fields]) OR "high blood pressure"[All Fields])) AND ((clinicaltrial[Filter] OR randomizedcontrolledtrial[Filter]) AND (2010:2021[pdat]))

Translations

Nursing: "nursing"[MeSH Terms] OR "nursing"[All Fields] OR "nursings"[All Fields] OR "nursing"[Subheading] OR "nursing's"[All Fields]

Interventions: "intervention's"[All Fields] OR "interventions"[All Fields] OR "interventive"[All Fields] OR "methods"[MeSH Terms] OR "methods"[All Fields] OR "intervention"[All Fields] OR "interventional"[All Fields]

Telehealth: "telehealth's"[All Fields] OR "telemedicine"[MeSH Terms] OR "telemedicine"[All Fields] OR "telehealth"[All Fields]

high blood pressure: "hypertension"[MeSH Terms] OR "hypertension"[All Fields] OR ("high"[All Fields] AND "blood"[All Fields] AND "pressure"[All Fields]) OR "high blood pressure"[All Fields]

Limits: Clinical Trail. RCT, 2010-2021

**Scopus**

Search

Results: 867

nursing AND interventions AND telehealth AND high AND blood AND pressure

**Cochrane Database**

Search

Results:14

Nursing interventions AND Telehealth AND high blood pressure

**Cinahl**

Search

Results: 2

nursing interventions AND ( telehealth or telemedicine ) AND ( high blood pressure or hypertension )

**ProQuest**

Search

Resuls: 5

Telemedicine AND Hypertension

**WOS**

Search

Results: 20

Nursing Interventions AND Telehealth OR Telemedicine AND high blood pressure
