# Supplementary material for: Nurse-led telehealth intervention effectiveness on reducing hypertension: a systematic review
Source: BMC Nurs. 2023 Jan 17;22:19. doi: 10.1186/s12912-022-01170-z (PMC9843665; doi:10.1186/s12912-022-01170-z)
Supplement: Supplementary file 3 — Additional file 3: Appendix 3: Critical appraisal results for included studies. [file 12912_2022_1170_MOESM3_ESM.docx]

**Appendix 3: Critical appraisal results for included studies**

| Author | *Q1* | *Q2* | *Q3* | *Q4* | *Q5* | *Q6* | *Q7* | *Q8* | *Q9* | *Q10* | *Q11* | *Q12* | *Q13* | *Total* |
| --- | --- | --- | --- | --- | --- | --- | --- | --- | --- | --- | --- | --- | --- | --- |
| Wakefield et al (2011) | Y | Y | Y | N | N | Y | Y | Y | Y | Y | Y | Y | Y | (85%) |
| Cicolini et al (2013) | Y | Y | Y | N | Y | Y | Y | Y | Y | Y | Y | Y | Y | (92%) |
| Brennan et al (2010) | Y | Y | Y | Y | N | N | Y | Y | Y | Y | Y | Y | Y | (85%) |
| Myoungsuk (2019) | Y | Y | Y | Y | Y | Y | Y | U | Y | Y | Y | Y | Y | (92%) |
| Hebert et al (2011) | Y | Y | Y | N | N | Y | Y | Y | Y | Y | Y | Y | Y | (85%) |
| Bosworth et al (2011) | Y | Y | Y | N | N | Y | Y | Y | Y | Y | Y | Y | Y | (85%) |

| Citation | *Q1* | *Q2* | *Q3* | *Q4* | *Q5* | *Q6* | *Q7* | *Q8* | Total |
| --- | --- | --- | --- | --- | --- | --- | --- | --- | --- |
| Choi and Kim (2014) | Y | N | Y | Y | Y | Y | Y | Y | (88%) |

Y= Yes

N= No

U= Unclear
